# Complete genome sequence of a novel toti-like virus from the plant-pathogenic oomycete *Phytophthora cactorum*

**DOI:** 10.1007/s00705-020-04642-2

**Published:** 2020-05-04

**Authors:** Anna Poimala, Eeva J. Vainio

**Affiliations:** Natural Resources Institute Finland (Luke), Natural Resources Unit, Forest Health and Biodiversity, Latokartanonkaari 9, 00790 Helsinki, Finland

## Abstract

This report describes the complete genome sequence of a double-stranded RNA (dsRNA) virus infecting the oomycetous plant pathogen *Phytophthora cactorum*. The virus genome consists of a single dsRNA segment of 5699 bp with two open reading frames predicted to overlap with each other and encoding a putative capsid protein of 705 aa and an RNA-dependent RNA polymerase of 779 aa. Sequence comparisons indicated that this virus, designated as “Phytophthora cactorum RNA virus 1” (PcRV1), shares the highest sequence similarity with the unclassified Pythium splendens RNA virus 1 (58% RdRp aa sequence identity). Phylogenetic analysis revealed that these two oomycete viruses group together with Giardia lamblia virus (GVL; family *Totiviridae*) and several unclassified toti-like viruses from arthropods, fish and fungi. This is the first report of a toti-like virus in a member of the genus *Phytophthora* and the first virus characterized in *P. cactorum.*

*Phytophthora cactorum* (Leb. and Cohn) Schröeter is an oomycetous phytopathogen of the Kingdom Stramenopila that causes root, collar and crown rot as well as foliar and fruit infections on a broad range of hosts, including over 200 species of trees, ornamentals, and fruit crops [5]. On *Betula* spp., *P. cactorum* is mostly a problem in nurseries, causing stem lesions on seedlings [13]. Prior to this report, no viruses had been described in *P. cactorum*, whereas a few viruses are known in congeneric host species: alphaendornaviruses infect members of the *Phytophthora* taxon ‘douglasfir’ and *P. ramorum* [9], and four dsRNA viruses have been described in the potato late blight pathogen *P. infestans* [2]. Two of these four are not closely related to other known viruses but have some resemblance to members of the family *Astroviridae*, whereas one is affiliated with a family-level group tentatively called ‘fusagraviruses’ and one is an unclassified member of the family *Narnaviridae*. In this study, we report the complete genome sequence of a new toti-like virus infecting *P. cactorum*, designated as "Phytophthora cactorum RNA virus 1" (PcRV1)*.*

## Provenance of the virus material

**Fig. 1:**
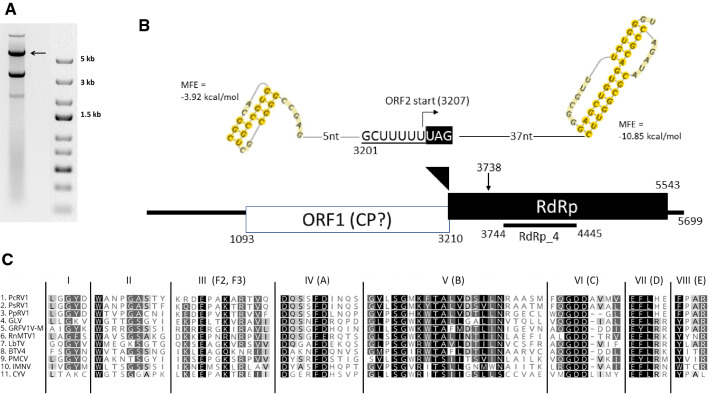
**A.** dsRNA profile of *Phytophthora cactorum* isolate BirchT_KT09 visualized in 1% agarose after 90-min electrophoresis at 120 V. PcRV1 is indicated by an arrow, while the other dsRNA elements present in the same host isolate will be reported elsewhere. The amount of O’GeneRuler Express DNA Ladder (Thermo Scientific) was 0.5 µg. According to the manufacturer, the 1.5-kb band corresponds to 100 ng of DNA per 0.5 ug of ladder. **B.** Schematic representation of the genomic organization of PcRV1. The open reading frames (ORFs) and the untranslated regions (UTRs) are indicated by open bars and single lines, respectively. The underlined sequence shows a putative slippage site (shifty heptamer), and the putative H-type pseudoknots are shown 37 nt to the right and 5 nt to the left. The first AUG of ORF2 is at nt position 3738. **C.**. Sequence comparison of the eight conserved motifs [1] in the RdRps of PcRV1 and other representative viruses of the family *Totiviridae.* Colors indicate aa similarity. Black, 100% similar; dark grey, 80–100% similar; light grey, 60–80% similar; white, less than 60% similar. Virus name abbreviations are the same as in the dendrogram (Fig. 2)

*Phytophthora cactorum* isolate BirchT_KT09 was isolated in 2009 from a trunk lesion on silver birch (*Betula pendula* Roth.) in Denmark by Kirsten Thinggaard, cultivated on 2% malt extract agar plates, and identified based on its morphology and ribosomal internal transcribed spacer 2 (ITS 2) sequence. Total dsRNA was extracted from 2.5 g of cultivated mycelia using cellulose affinity chromatography [12]. A 5.7-kb dsRNA element was purified from an agarose gel (Fig. 1A), and cDNA synthesis was conducted using tagged random hexamer priming followed by PCR amplification as described by Márquez et al. [11] except that Maxima H- Reverse Transcriptase and DyNAzyme II DNA polymerase (Thermo Scientific) were used. The amplicons were cloned using a TOPO TA Cloning Kit, (Thermo Scientific) and sequenced by Macrogen Inc., after which specific primers for amplification of the whole sequence were designed (data not shown). The whole sequence was determined based on direct Sanger sequencing of overlapping PCR amplicons (< 1000 bp with DreamTaq DNA polymerase and > 1000 bp with Phusion High-Fidelity DNA Polymerase, Thermo Scientific), sequencing the products of at least two separate PCR reactions to cover each nucleotide position. The terminal sequences were determined using T4 RNA adapter ligation followed by PCR amplification with specific primers and Phusion High-Fidelity DNA Polymerase (Thermo Scientific). Sequence data were analyzed using Geneious R10 (Biomatters Ltd., New Zealand). Sequence alignment for phylogenetic analysis was conducted using MAFFT v7.388 and the Blosum45 substitution matrix. Phylogenetic analysis was carried out using the MrBayes program implemented in Geneious R10 with the following parameters: rate matrix LG + *G* + *I* with five gamma categories; 1.1 × 10^6^ cycles for the MCMC algorithm, sampling one tree per 200 cycles; discarding 10^5^ samples as burn-in.

## Sequence properties

The complete genome sequence of PcRV1 (isolate PcRV1_BpKT09, GenBank accession number MN956531) consists of a single segment of 5,699 bp (Fig. 1A) with a 55% G + C content. The genome has a 1092-nt-long UTR at the 5′ terminus and a 156-nt UTR at the 3′ terminus (Fig. 1B). The genome is predicted to encode two large ORFs. ORF1 (705 aa; nt positions 1,093–3,210) shares 50.85% aa BLASTx identity (query coverage, 99%) with the putative coat protein of Pythium splendens RNA virus 1 (PsRV1; BBJ21454), as well as 37.80% and 29.72% BLASTx identity with putative CP sequences named as “Plasmopara viticola associated totivirus-like 1 and 2”, respectively (unpublished; GenBank accession numbers MN545914-5). Except for these three viruses, the predicted ORF1 did not share any BLASTp or BLASTx similarity with known proteins, and no putative conserved domains were detected with NCBI conserved domain search.

A shifty heptamer motif (GCUUUUU; nt positions 3,201-3,207), which may facilitate − 1 programmed ribosomal frameshifting in virus transcripts, was observed at the end of ORF1 (Fig. 1A). Based on this, the second ORF is predicted to begin with leucine in the shifty heptamer motif at 3,207 nt, overlapping in the -1 frame with ORF1. ORF2 (779 aa; nt positions 3,207–5,543) codes for a putative RNA-dependent RNA polymerase (RdRp) and shares the highest BLASTx identity (58.64%; query coverage, 99%) with PsRV1 RdRp (LC467965). No H-type RNA pseudoknot, which could stimulate frameshifting, was detected next to the slippery sequence, although a pseudoknot was predicted at a 37-nt distance from the ORF1 stop codon using HPknotter [4] (Fig. 1B). The ORF2 sequence includes a conserved region resembling members of the pfam02123 protein family, including RdRps from members of the genera *Luteovirus, Totivirus* and *Rotavirus* (aa residues 180–501). The genome organization of PcRV1 corresponds to that found in members of the family *Totiviridae* [6], where the 5′-proximal ORF encodes the CP and the 3′-proximal ORF encodes the RdRp. Based on this, we propose that ORF1 encodes a CP also in PcRV1. However, due to the high level of sequence divergence, the putative CP was not used for further phylogenetic inference.

**Fig. 2:**
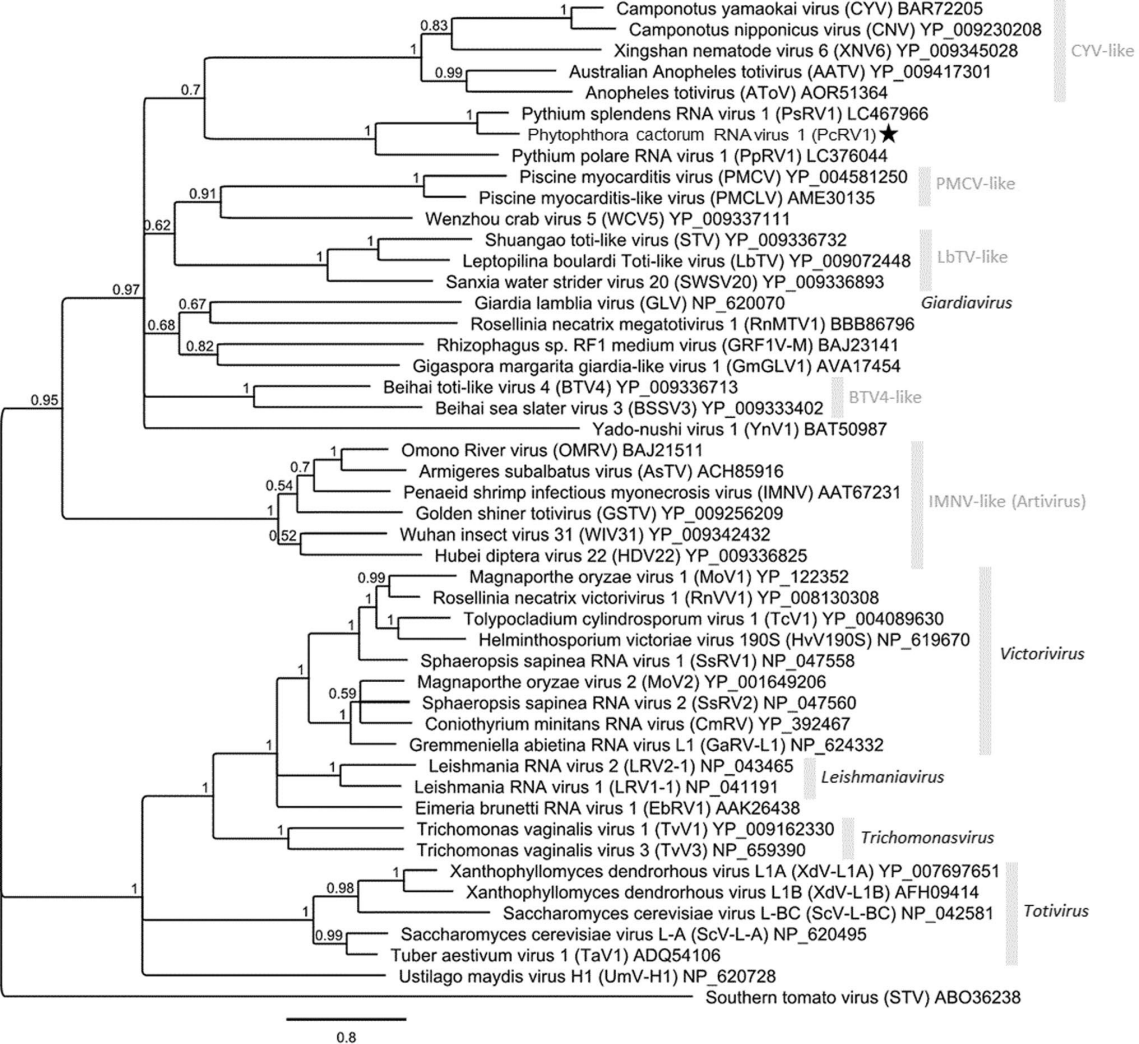
Phylogenetic analysis based on the RdRp amino acid sequences of PcRV1 and those of related viruses including representatives of the five *Totiviridae* genera as well as other related phylogenetic groups awaiting classification [14]. The sequence region included in the MAFFT alignment contained the RdRp conserved motifs I-VIII and consisted of 492 aa sites. The analysis was conducted with MrBayes, using southern tomato virus as an outgroup. The scale bar shows 0.8 aa substitutions per site, and percentage posterior probabilities are shown at branch nodes

Viruses resembling Giardia lamblia virus (GLV), the sole classified member of the genus *Giardiavirus*, family *Totiviridae* (ICTV Master Species List 2018b.v2 at https://talk.ictvonline.org/), have recently been detected in arthropods, fish and fungi [4, 10, 14]. Phylogenetic analysis based on RdRp sequences (Fig. 2) showed that PcRV1 and the *Pythium* viruses PsRV1 and Pythium polare RNA virus 1 (PpRV1) form a highly supported clade and cluster among the new GLV-like viruses. Interestingly, Shiba et al. [15] detected a virus-like RdRp sequence similar to that of PsRV1 in the transcriptome of *Heterosiphonia pulchra*, which also included transcripts related to *Phytophthora cactorum*. Thus, the natural sample of *H. pulchra* might have included an associated *P. cactorum* hosting a similar toti-like virus. The virus-like sequence from *H. pulchra* showed 37.0% aa sequence identity to the PcRV1 RdRp based on global alignment. Among the GLV-like viruses, the presence of a shifty heptamer motif together with the absence of a nearby RNA pseudoknot is also found in piscine myocarditis virus (PMCV) and Camponotus yamaokai virus (CYV) [14]. In addition, Jamal et al. [7] did not detect the CP-RdRp fusion product of Alternaria alternata victorivirus 1 (AalVV1), whose genome also has a similar structure, suggesting that the translation of AalVV1 RdRp might involve proteolytic processing or a novel type of termination-coupled reinitiation mechanism.

According to the virus species demarcation criteria for the genus *Totivirus* in the  family *Totiviridae* [8], viruses found only in distinct host species are for that reason considered members of different species, and less than 50% sequence identity at the protein level generally reflects a species difference. The RdRp sequence identity between PcRV1 and the *Pythium* virus PsRV1 exceeds this threshold but remains moderate (ca. 58%) and corresponds to the 60% maximum identity criterion set for different species of the genus *Victorivirus* in family *Totiviridae.* In addition, the hosts represent different oomycete genera. Therefore, we propose that these viruses should be considered members of different species. As more related species are described, the ICTV may need to adjust the species demarcation criteria for this virus group accordingly. It should be noted that strains of *Pythium* and *Phytophthora* often co-occur in soil and diseased plants, which could provide a potential route for interspecies virus transmission. However, no mechanisms for interspecies virus transmission between members of oomycete genera have been described thus far, and in the case of *Phytophthora infestans,* even intraspecies transmission of viruses between somatically incompatible isolates has been shown to be unsuccessful [3].
